# Correlation between effective dose and radiological risk: general concepts*

**DOI:** 10.1590/0100-3984.2014.0097

**Published:** 2016

**Authors:** Paulo Roberto Costa, Elisabeth Mateus Yoshimura, Denise Yanikian Nersissian, Camila Souza Melo

**Affiliations:** 1 PhD, Professor at the Instituto de Física da Universidade de São Paulo (IFUSP), São Paulo, SP, Brazil.; 2 PhD, Full Professor at the Instituto de Física da Universidade de São Paulo (IFUSP), São Paulo, SP, Brazil.; 3 PhD, Physicist at the Instituto de Física da Universidade de São Paulo (IFUSP), São Paulo, SP, Brazil.; 4 Specialist in Physics of Diagnostic Radiology, Physicist at the Instituto de Física da Universidade de São Paulo (IFUSP), São Paulo, SP, Brazil.

**Keywords:** Radiation dosage, Radiation effects, Diagnostic imaging/adverse effects, Relative biological effectiveness

## Abstract

The present review aims to offer an educational approach related to the limitations in the use of the effective dose mgnitude as a tool for the quantification of doses resulting from diagnostic applications of ionizing radiation. We present a critical analysis of the quantities accepted and currently used for dosimetric evaluation in diagnostic imaging procedures, based on studies published in the literature. It is highlighted the use of these quantities to evaluate the risk attributed to the procedure and to calculate the effective dose, as well as to determine its correct use and interpretation.

## Abstract (pt)

Este trabalho de revisão pretende oferecer uma abordagem educacional relacionada às limitações na utilização da grandeza dose efetiva como ferramenta para quantificação de doses decorrentes de aplicações em diagnóstico médico utilizando radiações ionizantes. Os autores apresentam uma análise crítica sobre as grandezas aceitas e utilizadas atualmente para a avaliação dosimétrica em procedimentos de diagnóstico médico por imagem, tendo como base estudos publicados na literatura. Destacam-se as formas de utilização dessas grandezas para a avaliação do risco atribuído ao procedimento e para o cálculo da dose efetiva e sua correta utilização e interpretação.

## INTRODUCTION

Much of the motivation in establishing dosimetric methods related to diagnostic imaging procedures is based on the interest in estimating health risks to a patient subjected to a given type of examination using ionizing radiation. This motivation has a strong correlation with the need to balance the risks and benefits of a new diagnostic modality or with the desire to ensure that the chosen modality is the one that will have the fewest potentially harmful effects on the health of the patient.

This work presents a critical analysis of the quantities accepted and currently used for dosimetric evaluation in medical diagnostic imaging procedures, based on studies published in the literature. Emphasis will be given to the ways in which these quantities are used in assessing the risk attributed to a given procedure and in calculating the effective dose. The correct use and interpretation of the effective dose are also addressed.

## EFFECTIVE DOSES AND RADIATION RISKS

The method presented by the International Commission on Radiation Protection (ICRP) to calculate the effective dose is based on the use of multiplicative models of risk factors, applied to quantities that are more fundamental, such as the absorbed dose^(1)^. Behind the risk factors published by the ICRP is the opportunity to use the knowledge associated with the long-term biological effects of ionizing radiation, knowledge accumulated since the beginning of last century^(2)^, to draw a correlation between exposure to radiation in living beings and the biological effects associated with such exposure. Using the concepts of stochastic effects, Drexler et al.^(3)^ made a detailed interpretation of this quantity, also known as the "effective dose equivalent". The authors predicted its association with doses in small groups or even in individuals. The authors made it clear that, in those cases, it is not possible to calculate the true risks or detriment using the factors presented, stating that the data available in the official radiological protection publications of the period are applicable to the average population of workers and that the detriment rates for other populations would, of course, be different. They also stated that the use of effective dose is justified in order to follow the principle of limitation of occupationally exposed individuals, considering uniform whole-body exposures. Although the effective dose concept can be applied in situations of non-uniform irradiation, the results derived from models for this kind of situation cannot be associated with the basic radiological protection quantities.

Drexler et al.^(4)^ stated that applying the concepts of effective dose to the radiological protection of patients is misleading and meaningless when used in order to estimate individual or collective patient risk. The authors discussed the use of this quantity to support the optimization procedures, to compare risks between methods and to set dose constraint levels, as well as to estimate risks to individuals and populations undergoing diagnostic imaging procedures. Based on that argument, they demonstrated support for their previous interpretation, concluding that the use of effective dose is incorrect because of inappropriate simplification of the underlying biological mechanisms and the inadequacy of using weighting factors connected to the definition of the effective dose for a given patient population.

A number of studies associating the quantity effective dose with diagnostic imaging procedures have been published in widely circulated journals. Such associations have been identified in studies related to imaging tests in which there is a possibility that high doses will be used, such as interventional radiology^(5)^ and computed tomography (CT) scans^(6)^, as well as other modalities^(7)^.

## CALCULATING AND INTERPRETING THE QUANTITY EFFECTIVE DOSE

According to national^(8)^ and international^(1)^ regulations, the effective dose can be calculated by determining the sum of the weighted equivalent doses in different organs and tissues:

E=ΣTwTHT=ΣTΣRwTwRDT.Rphotons⇒E=ΣTwTDT1

where *H_T_* and *D_T,R_* represent the equivalent dose in tissue or organ *T* and the mean absorbed dose in tissue or organ *T* from radiation *R*, respectively; *w_T_* is the tissue weighting factor; and *w_R_* is the radiation weighting factor for radiation *R*^(9)^.

According to ICRP Publication 103^(1)^, the fact that the effective dose and the equivalent dose in an organ or tissue are not directly measurable quantities must be taken into account. Therefore, operational quantities have been defined. Those quantities are obtained through the use of instruments^(10)^ to determine the radiation protection quantities for the assessment of occupational exposures.

The numerical simulator of the human body (computational phantom) recommended by the ICRP for determining the conversion factors is based on Zankl et al.^(11-13)^. On the basis of the correlation between the voxels provided by these computer models and the organs of the human body, adjustments were made in order to render these models suitable in relation to the masses of the organs in the reference male and female, as defined in ICRP Publication 89^(14)^. These reference phantoms were adopted in order to determine the conversion factors reproduced in ICRP Publication 103, which correlate physical quantities-such as the air kerma, the particle fluence induced by external radiation and the activity incorporated for internal exposure-with the radiological protection quantities such as the equivalent dose and the effective dose.

Paragraph B132 of ICRP Publication 103^(1)^ details the method of obtaining the effective dose, regardless of patient gender. This method was used in order to define unified conversion factors and can be used, simplistically, for radiation protection purposes. The document shows that the effective dose is calculated on the basis of the equivalent doses obtained for organs and tissues, through the use of the following equation:

$$E=\Sigma_{T}w_{T}\left[\frac{H_{T}^{M}+H_{T}^{F}}{2}\right]\;\;\;\;\;\;\;\;\;\;\;\;\;\left(2\right)$$

where *H_T_^M^* and *H_T_^F^* are the equivalent doses obtained for organs and tissues of the reference male and the reference female, respectively.

**Figure 1:**
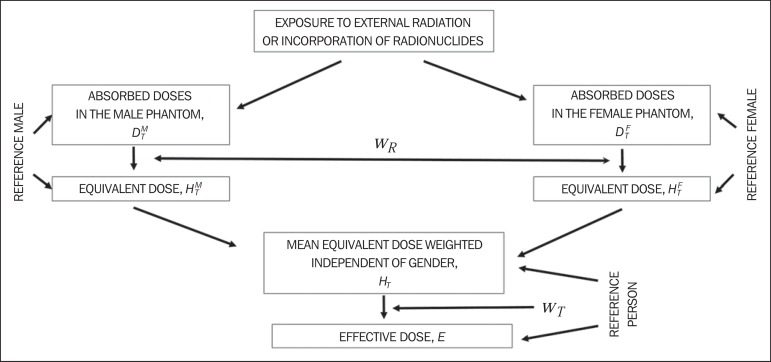
Method adopted by the ICRP to determine the gender-specific independent effective dose through the use of weighted average values for the reference person. (Based on ICRP Publication 103^(1)^).

As a result of the independent gender mean represented by Eq. (2), the quantity effective dose, for radiation protection purposes, provides values that take into account the exposure conditions of the reference person, rather than those of a specific individual. When using the quantity defined by the ICRP, the fact that the weighting factors are obtained from average values derived from a large number of individuals of both genders should be taken into account. Figure 1 shows the ICRP method adopted to determine the effective dose regardless of gender, by means of weighted mean values for the reference person.

In 2000, before the publication of ICRP Publication 103^(1)^, McCollough et al.^(15)^ published an important contribution to the interpretation of the quantity effective dose and the methods for its calculation. The authors described the various definitions of that quantity presented in different ICRP Publications issued since 1977. They also stated that the values corresponding to the application of the weighting factors and the association with the resulting overall detriment are relevant only to the population and the irradiation conditions from which those factors were derived. Thus, according to the interpretation of Drexler et al.^(3,4)^, they reaffirm that the detriment resulting from the application of the concept of effective dose only makes sense for the population in general or for specific populations of workers, not being suitable for use to patients, due to differences related to age and gender. Finally, the authors highlighted the inconsistency related to the use of weighting factors, because the normalization cannot be applied in cases of inhomogeneous radiation.

However, one should not underestimate the importance of establishing methods for estimating risks related to medical exposure. According to McCollough et al.^(15)^, the most comprehensive approach to risk assessment is to increase the knowledge of the doses to all relevant organs and the risks for those organs, associated with and related to age and gender. The important point of this discussion is the correct definition of the applicability limits of the weighting factors set by the ICRP for dose-risk correlation in medical procedures that use ionizing radiation. Therefore, the effective dose can be used in order to compare the relative detriments between different radiologic procedures and to facilitate the comparison of detriments in the application of different radiation sources. However, in either case, it must be calculated for patient populations of consistent age and gender.

Despite of the limitations described above, there are many recent studies correlating effective doses with diagnostic imaging procedures^(16-18)^. Not all of those publications addressed the limitations, uncertainties, and adaptation of interpretation that should be associated with the quantification of effective dose or the fact that it cannot be associated with the risk to individuals^(19)^.

Verdun et al.^(20)^ reviewed the concepts and quantities of radiological protection. The authors explained that the quantity effective dose has a stochastic origin and correlates with health risks as follows: "This quantity takes the health risk (fatal and nonfatal cancers, taking into account the latency period as well as severe hereditary disorders) of a "standard" patient who is nonuniformly exposed to ionizing radiation and transposes it into a situation in which this patient would be uniformly exposed to a radiation field. This methodology is used for monitoring workers exposed to ionizing radiation." The concept of the standard patient is presented in Chapter 7 of ICRP Publication 103^(1)^, which states that the effective dose can be useful in cases of medical exposure, in order to draw comparisons between different procedures, between countries or hospitals in terms of the use of similar techniques and procedures, and between different types of equipment used in the same type of examination. This is valid provided that the reference patient or populations are homogeneous with respect to age and gender. The publication emphasizes that the interpretation of the effective dose in relation to medical exposure is problematic when organs and tissues are partially exposed and when the exposure is heterogeneous.

In terms of the limitations related to the ICRP method for the determination of effective doses, Verdun et al.^(20)^ made it clear that, due to the differences in risk factors related to physical characteristics, age, and gender, the effective dose should not be used in order to infer excess relative risk to a particular individual^(21)^. Finally, the authors discouraged the use of these correlations for associated risks related to the use of radiation in diagnostic imaging procedures.

## CONTROVERSIES REGARDING THE USE OF EFFECTIVE DOSE IN DIAGNOSTIC IMAGING

The popularization and systematic use of the effective dose as a metric for dosimetric evaluation in medical procedures might have begun with the study conducted by Martin^(22)^, in 2007. In that study, the author presented a thoughtful reflection on the stochastic nature of the calculation of the detriment, which gives the weighting factors used in the calculation of effective dose, as well as on the uncertainties involved in its calculation. In particular, the author highlighted the excessive attention given to the effective dose calculations related to medical procedures and their associated risks, without due care in determining the explanations for those associations. The author also provided estimates that lead to uncertainty values of approximately 40% in calculating the effective dose for a standard patient. It is noteworthy that the use of the effective dose concept to estimate the risk for an individual subjected to a diagnostic imaging procedure is not recommended by the ICRP. Martin concluded that the effective dose is the only available quantity associated with the risk of a detriment to health. The author made it clear that the effective dose should not be related to an individual but rather to a non-gender-specific reference patient, for which the risks have been determined based on the average standard population. The author argued that, to calculate the risk for an individual patient, the best indicator should be related to the estimation of doses in all radiosensitive organs and tissues, as well as to the combination of those values with the specific risks to those organs, by age and gender.

Unquestionably, the great complexity that underlies the understanding of the quantities adopted for radiation protection^(23)^. Consequently, a great care should be taken when using this terminology.

The appropriateness of using the effective dose as the quantity adopted to represent doses related to exposures in patients or patient populations is a source of concern regarding its correct interpretation^(20)^ and of various controversies in the scientific community. Such disputes arise with the use of a measure originally introduced as a quantitative representation of the potential stochastic detriments, in particular cancer and hereditary effects, resulting from exposure of populations of workers and the general population^(24)^, as a representative measure of the dose received by patients undergoing radiological examinations.

Controversy regarding the effective dose led to a debate in the "point-counterpoint" section of the July 2010 issue of the Medical Physics journal^(25)^. In that article, Professor Borrás argued against the use of the effective dose as a representative measure of doses during medical procedures, whereas Professor Huda argued in favor of such use. Professor Huda was also the protagonist of another debate, this time with Dr. Cohen, presented in the 2011 issue of the journal Radiology^(26)^.

In Annex A of its 2008 report^(27)^, the United Nations Scientific Committee on the Effects of Atomic Radiation pointed out the numerous difficulties in obtaining reliable estimates of absorbed doses and consequently the effective doses corresponding to clinical examinations, noting that this quantity should always be correlated with homogeneous populations. That report highlighted three major approaches to estimating doses in patients undergoing radiological procedures: dose measurements directly in the patient; dose measurements in physical phantoms and Monte Carlo calculations. In each case, the associated uncertainties^(28)^ and difficulties were highlighted, as was the weak association that these results can have with estimates of risk or detriment.

McCollough et al.^(29)^ recently revisited the theme of the effective dose. The authors began by addressing the scenario in which a patient, after undergoing an examination, asks the doctor: "What dose did I receive?". The authors assert that what the patient really wants to know is how much risk is associated with the procedure performed. In addition, other medical professionals now have more access to information available in medical journals about the risk of stochastic effects resulting from procedures such as CT^(30)^.

Taking into account the limitations and uncertainties associated with the use of the effective dose to support the determination of risks from radiological procedures, McCollough et al.^(29)^ pointed out its usefulness as a tool for making comparisons between different types of radiation exposure and for converting the complex dose distribution in various tissues and organs into a single dosimetric parameter. The authors stated that the effective dose could be applied as a kind of "whole-body dose equivalent" value related to the risks arising from non-uniform irradiation in different diagnostic modalities. With this approach, it is possible to compare different dose values resulting from different imaging modalities with similar purposes, such as conventional coronary angiography, CT angiography and myocardial perfusion with nuclear medicine.

## THE IMPORTANCE OF PROPER INTERPRETATION OF THE QUANTITY EFFECTIVE DOSE

The article "Health risks from exposure to low levels of ionizing radiation: BEIR VII Phase 2"^(31)^, published in 2006, defined the effective dose as the sum of the doses absorbed by different organs from different types of radiation, multiplied by weighting factors for the organs and for the types of radiation. This definition is similar to that outlined in ICRP Publication 103^(1)^. The unit of the quantity effective dose is the sievert (Sv): 1 Sv = 1 J/kg. Excluding differences related to gender and age, equal effective doses correspond to approximately the same overall risk. For uniform whole-body exposure, even for a specific type of radiation, the effective dose is equal to the absorbed dose of radiation multiplied by the radiation weighting factor.

ICRP Publication 103 states that the effective dose is calculated for a reference person and not for an individual. In addition, ICRP Publication 116^(30)^ clarifies that the factors used to weight the absorbed doses to specific tissues do not vary with age or gender, and that the use of the weighted sum for obtaining the effective dose is not applicable to a specific individual. Therefore, the effective dose serves to support regulatory devices and comparative assessments of professional practices.

It should be noted that effective dose was established as a quantity applicable to planned situations. ICRP Publication 105^(33)^ states that the distribution of ages of workers and the general population can be significantly different from that of a population subjected to a given type of medical procedure that employs ionizing radiation. Other aspects, such as the gender of the patients undergoing the procedure, can be related to these age distributions, which are used in deriving the value of the effective dose.

The need to establish correlations between doses and the risks of performing radiological procedures can be questioned, because the principles of justification and optimization-known as the "as low as reasonably achievable" (ALARA) principle-have been properly applied and the risk/benefit ratio is acceptable. However, the convenience of using quantitative parameters that describe a procedure in terms of its potential risk to the health of patients, compared with the alternative practices or the application of a new technique in contrast with others currently in use, makes it a tempting option. For such correlations associated with the CT technique, Dixon^(34)^ emphasized the need to train all of the workers involved, which comprises adapting the instruction of medical students, technicians, technologists, and physicians of other specialties to include concepts regarding doses and their implications. The author also highlighted the apparent inconsistency of institutions that invest hundreds of thousands of dollars, euros, or Brazilian reals in the acquisition of a new CT modality but are reluctant to invest in training their staff, in the continuing education of their physicians, or in obtaining the support of medical physics professionals for the proper monitoring of aspects related to the dose in CT and in other modalities.

## CONCLUSIONS

The present review aims to provide readers with an educational approach related to limitations in the use of the effective dose quantity as a tool to access doses and health-related risks associated to diagnostic imaging procedures that employ ionizing radiation. This quantity can be used in order to compare the relative detriments of radiological procedures and other radiation sources when calculated for populations of patients that are homogeneous in terms of age and gender. The use of effective dose can also be useful for the purpose of comparisons between different procedures and techniques, or between different hospitals or countries provided that the reference patient or patient population are similar with respect to age and gender.

It should be borne in mind that the ICRP does not recommend using effective dose to estimate the risk for an individual subjected to a diagnostic imaging procedure. That is because the quantity was introduced as a means of representing the potential stochastic detriments resulting from the exposure of populations of workers and the general population. Therefore, it is incorrect to use effective dose as an estimator of individual risks for patients undergoing radio-logic studies.

In summary, the effective dose can be useful for estimating the detriment relative to non-uniform and partial-body irradiation; for optimizing radiological procedures involving multiple organs or tissues; for drawing comparisons between alternative procedures or background radiation levels; and for estimating the relative detriment attributed to multiple exposures or different modalities.

The effective dose serves to support regulatory documents and comparative assessments of professional practices, despite of the fact that its quantity is not able to represent the stochastic health risk resulting from exposures of a given worker or other individual.

For the medical community, the principle of justifying radiological procedures is more important than the determination of doses through the use of quantities that are not applicable to the estimation of risks^(35)^. It is understood that this is a factor, which affects radiation protection more significantly than does the determination of the individual dose.
